# Supplementary material for: Anti-Inflammatory Activity of Soluble Epoxide Hydrolase Inhibitors Based on Selenoureas Bearing an Adamantane Moiety
Source: Int J Mol Sci. 2022 Sep 14;23(18):10710. doi: 10.3390/ijms231810710 (PMC9501280; doi:10.3390/ijms231810710)

Anti-Inflammatory Activity of Soluble Epoxide Hydrolase Inhibitors Based on Selenoureas Bearing an Adamantane Moiety

Vladimir Burmistrov [1,2,*], Christophe Morisseau [1], Denis A. Babkov [3], Tatiana Golubeva [4,5], Dmitry Pitushkin [2], Elena V. Sokolova [3], Vladimir Vasipov [6], Yaroslav Kuznetsov [2], Sergey V. Bazhenov [7], Uliana S. Novoyatlova [7], Nikolay A. Bondarev [7], Ilya V. Manukhov [7], Victoria Osipova [8], Nadezhda Berberova [9], Alexander A. Spasov [3], Gennady M. Butov [2] and Bruce D. Hammock [1]

1 Department of Entomology and Nematology, Comprehensive Cancer Center, University of California, Davis, CA 95616, USA

2 Department of Chemistry, Technology and Equipment of Chemical Industry, Volzhsky Polytechnic Institute (Branch), Volgograd State Technical University, 404121 Volzhsky, Russia

3 Department of Pharmacology & Bioinformatics, Scientific Center for Innovative Drugs, Volgograd State Medi-cal University, 400131 Volgograd, Russia

4 Institute of Cytology and Genetics SB RAS, 630090 Novosibirsk, Russia

5 Specialized Educational Scientific Center, Novosibirsk State University, 630090 Novosibirsk, Russia

6 N.I. Vavilov All-Russian Institute of Plant Genetic Resources, 42, 44, Bolshaya Morskaya Street, 190000 St. Petersburg, Russia

7 Research Center for Molecular Mechanisms of Aging and Age-Related Diseases, Moscow Institute of Physics and Technology, Institutsky Lane 9, 141700 Dolgoprudny, Moscow Region, Russia

8 Toxicology Research Group of Southern Scientific Centre of Russian Academy of Science, 41 Chekhova Str., 344006 Rostov-on-Don, Russia

9 Department of Chemistry, Astrakhan State Technical University, 16 Tatisheva Street, 414056 Astrakhan, Russia

* Correspondence: crus_himself@mail.ru; Tel.: +7-905-338-2432

Supplementary Materials

NMR, Mass and IR spectra

NMR ^{1}H of compound 1

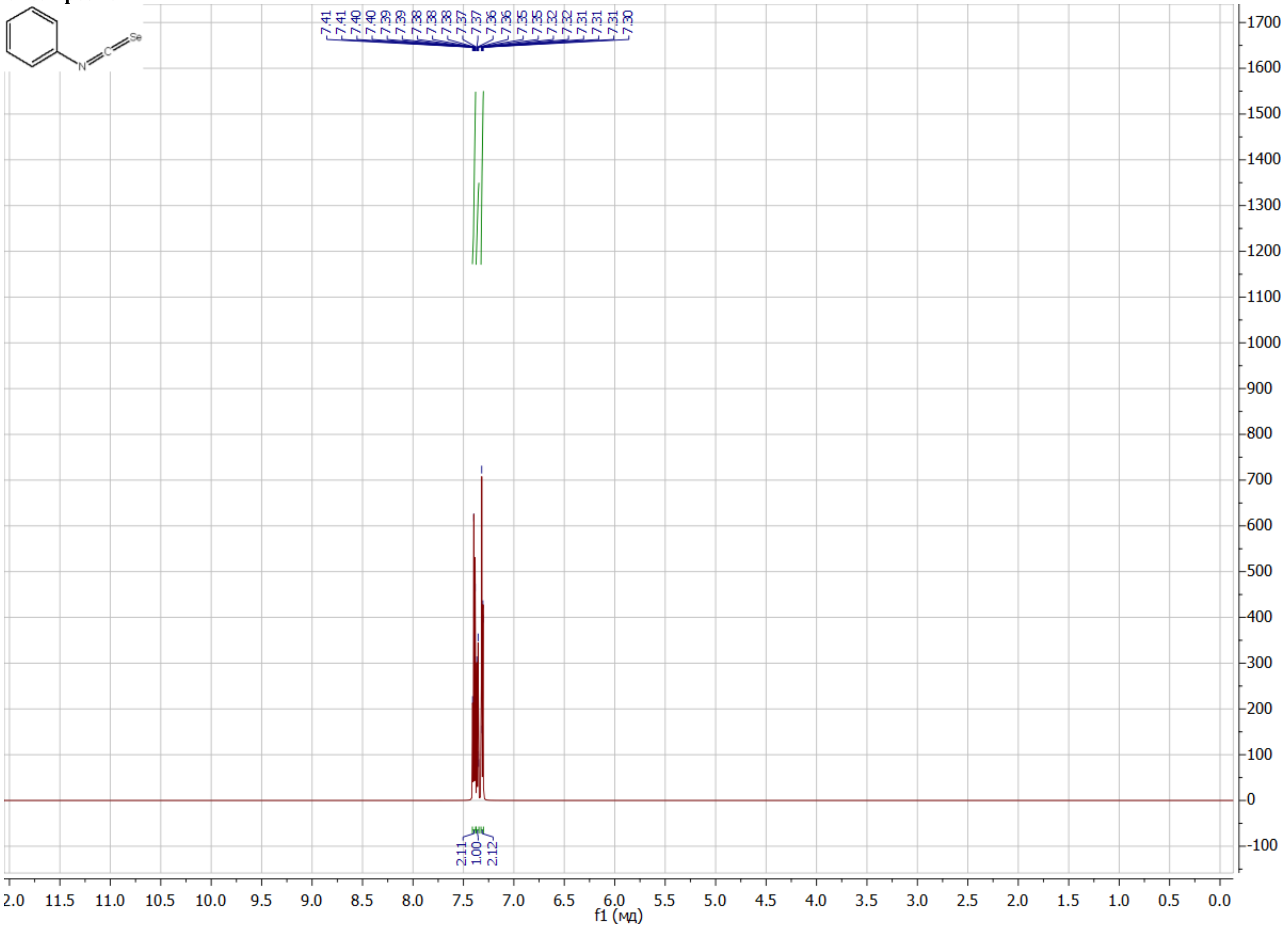

NMR ^{13}C of compound 1

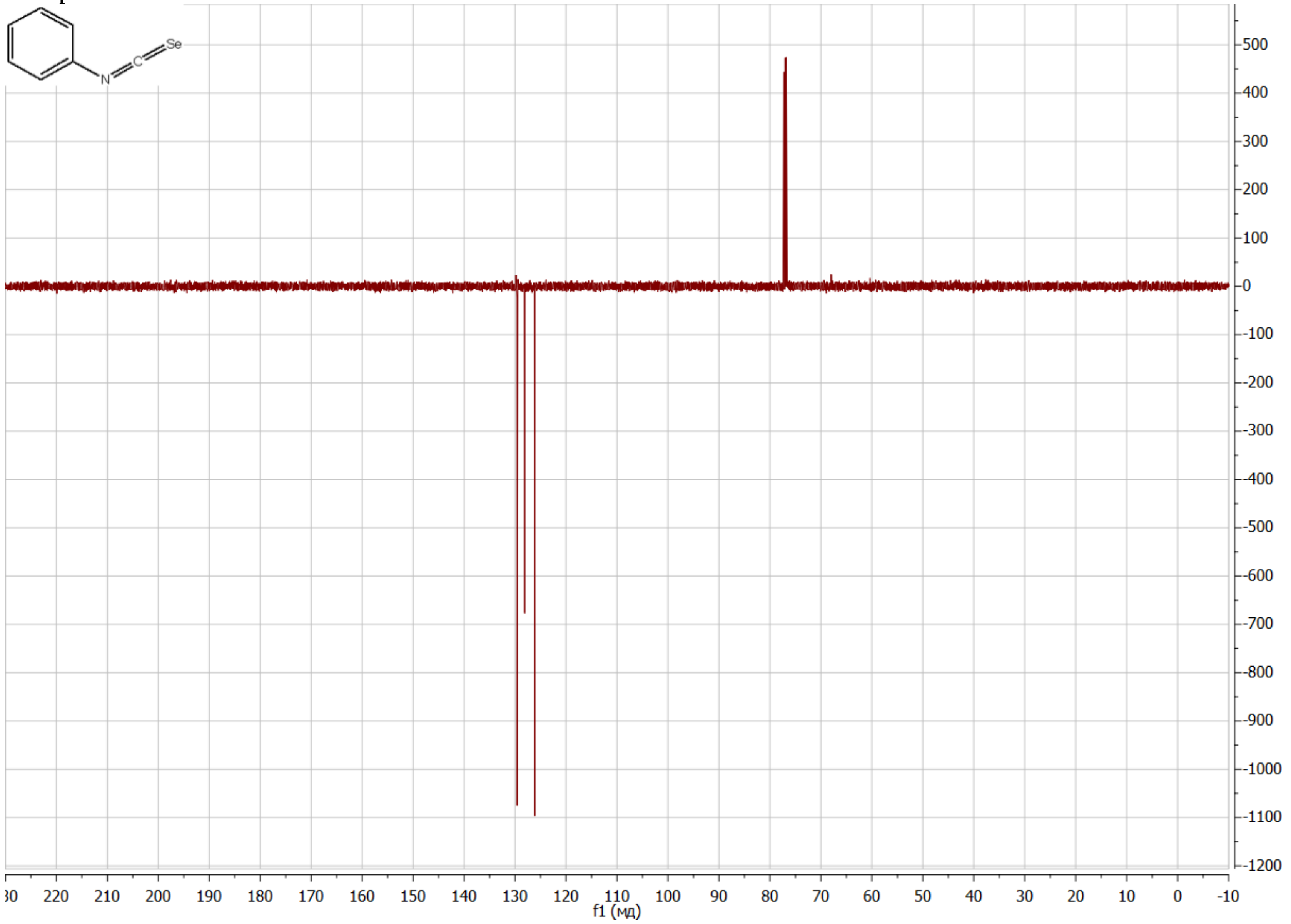

Chromatogram of compound 1

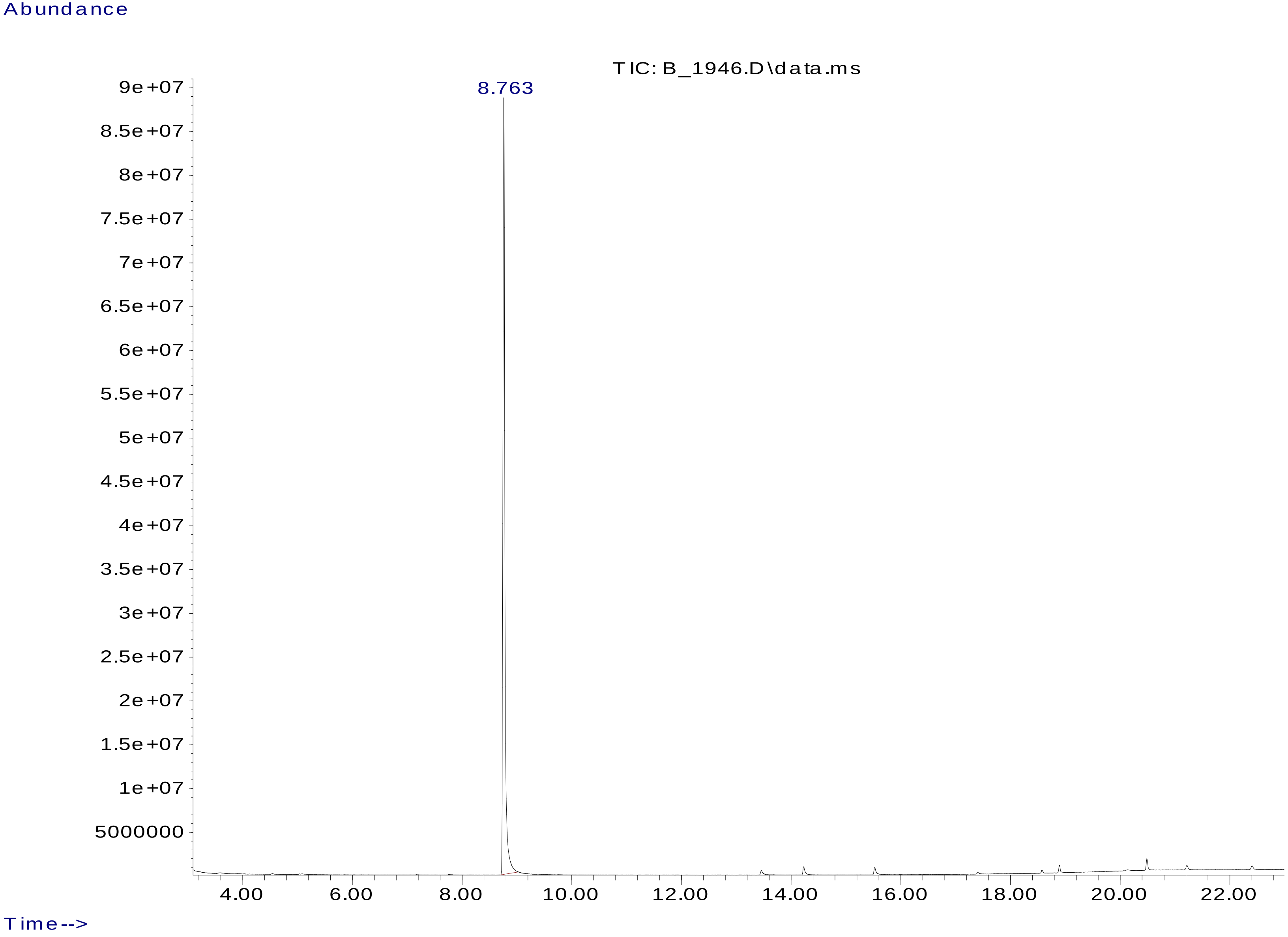

Mass specter of compound 1

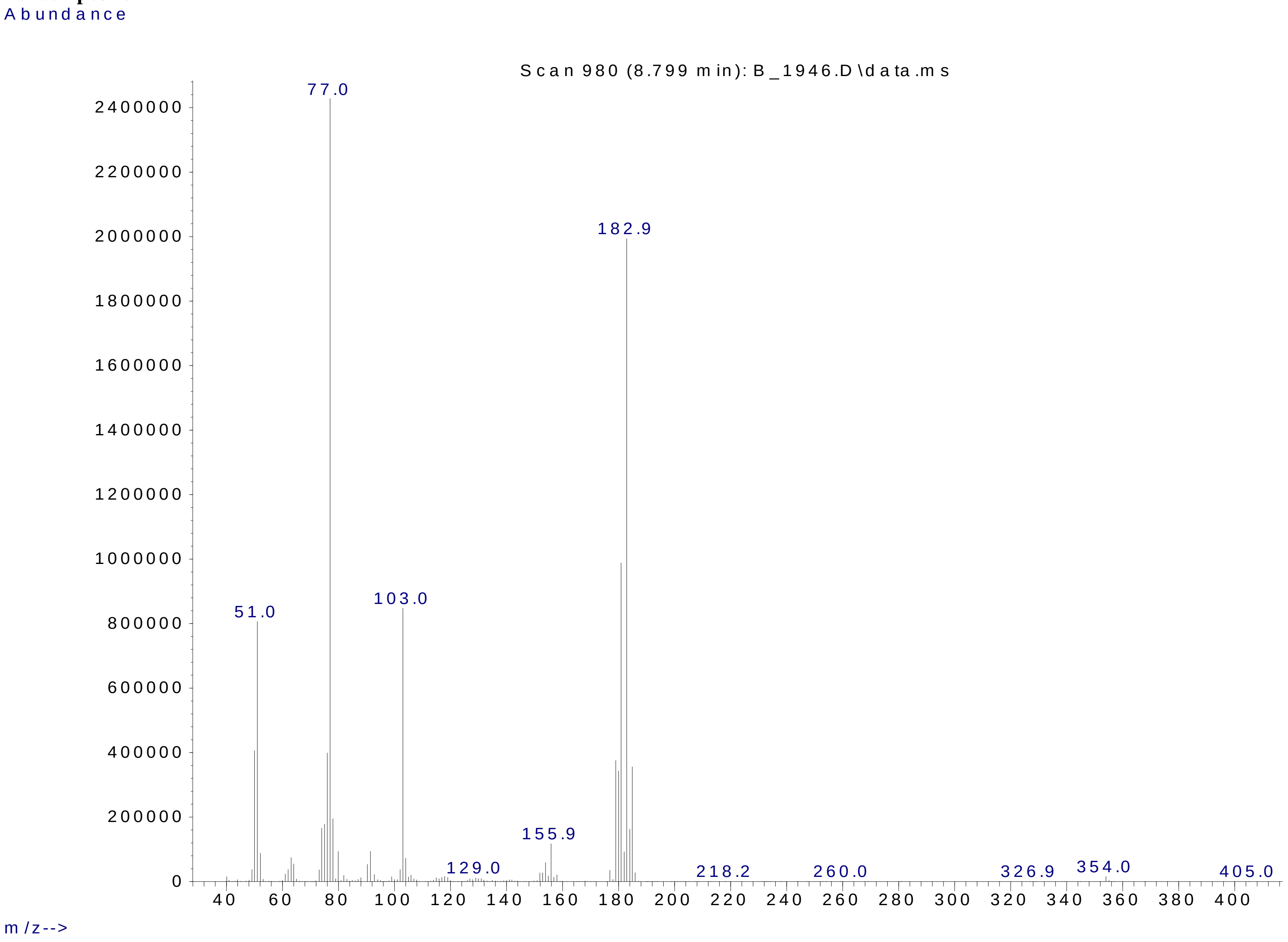

Chromatogram of compound 2

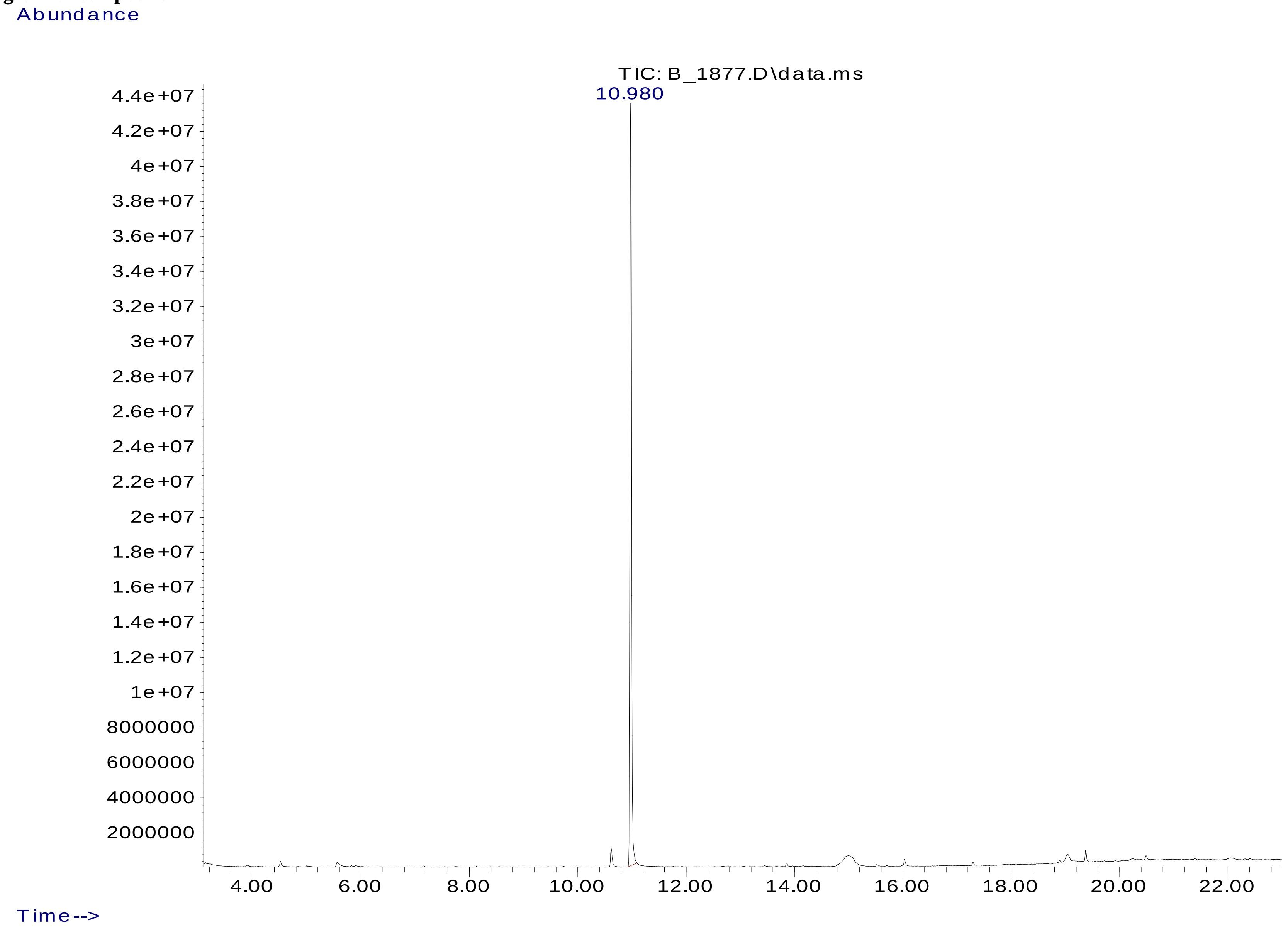

Mass specter of compound 2

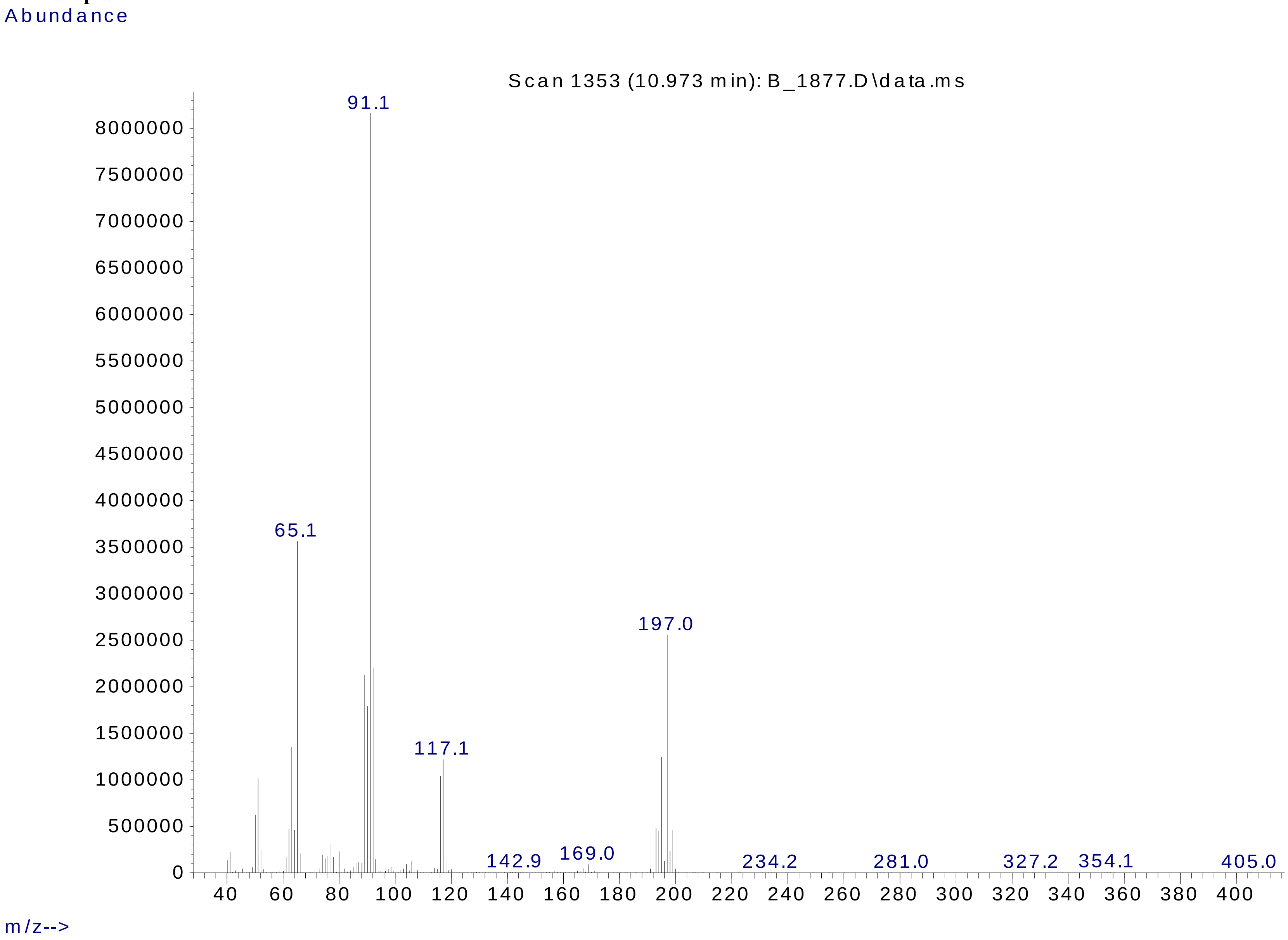

NMR ^{1}H of compound 4a

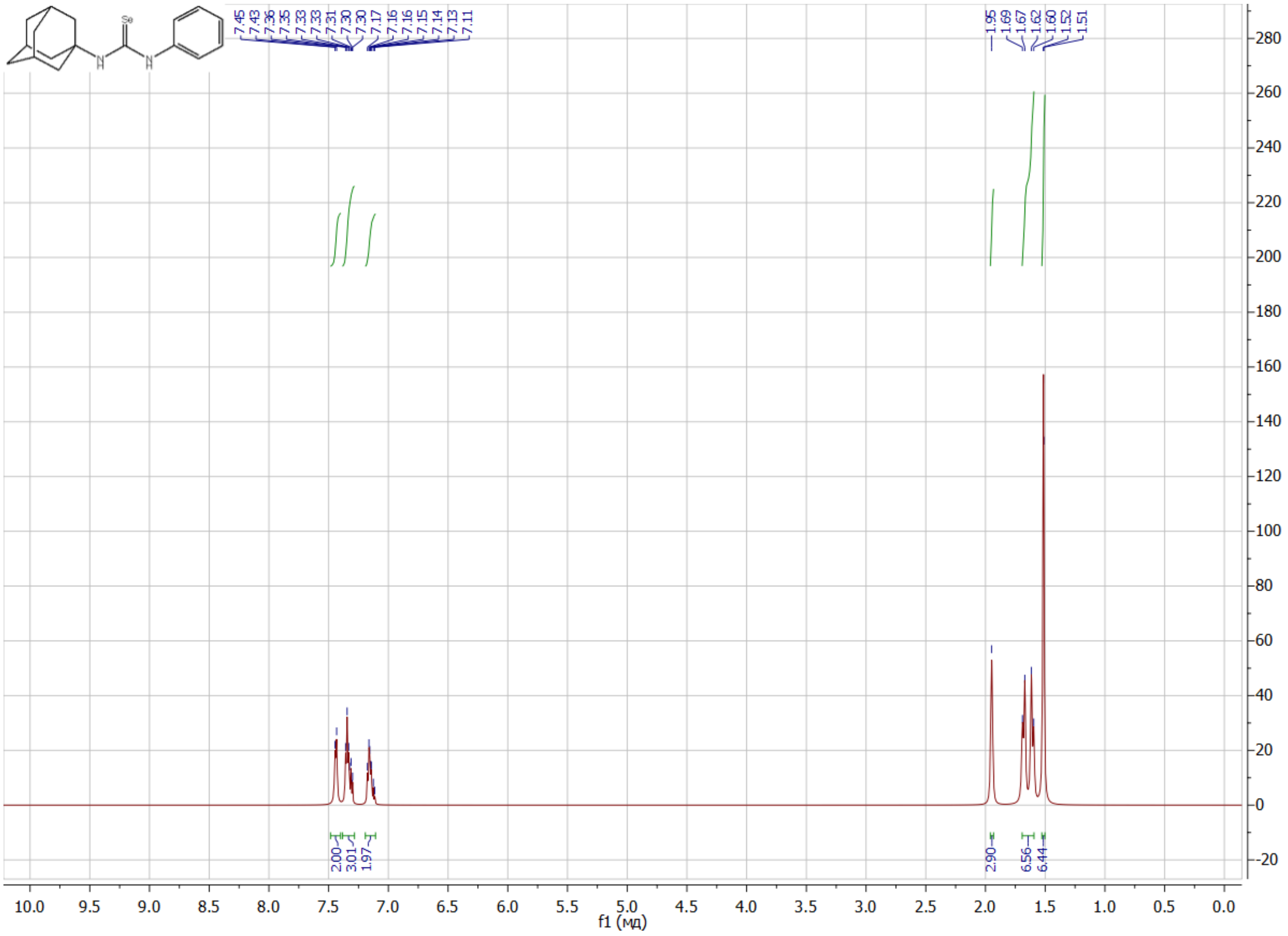

IR of compound 4a

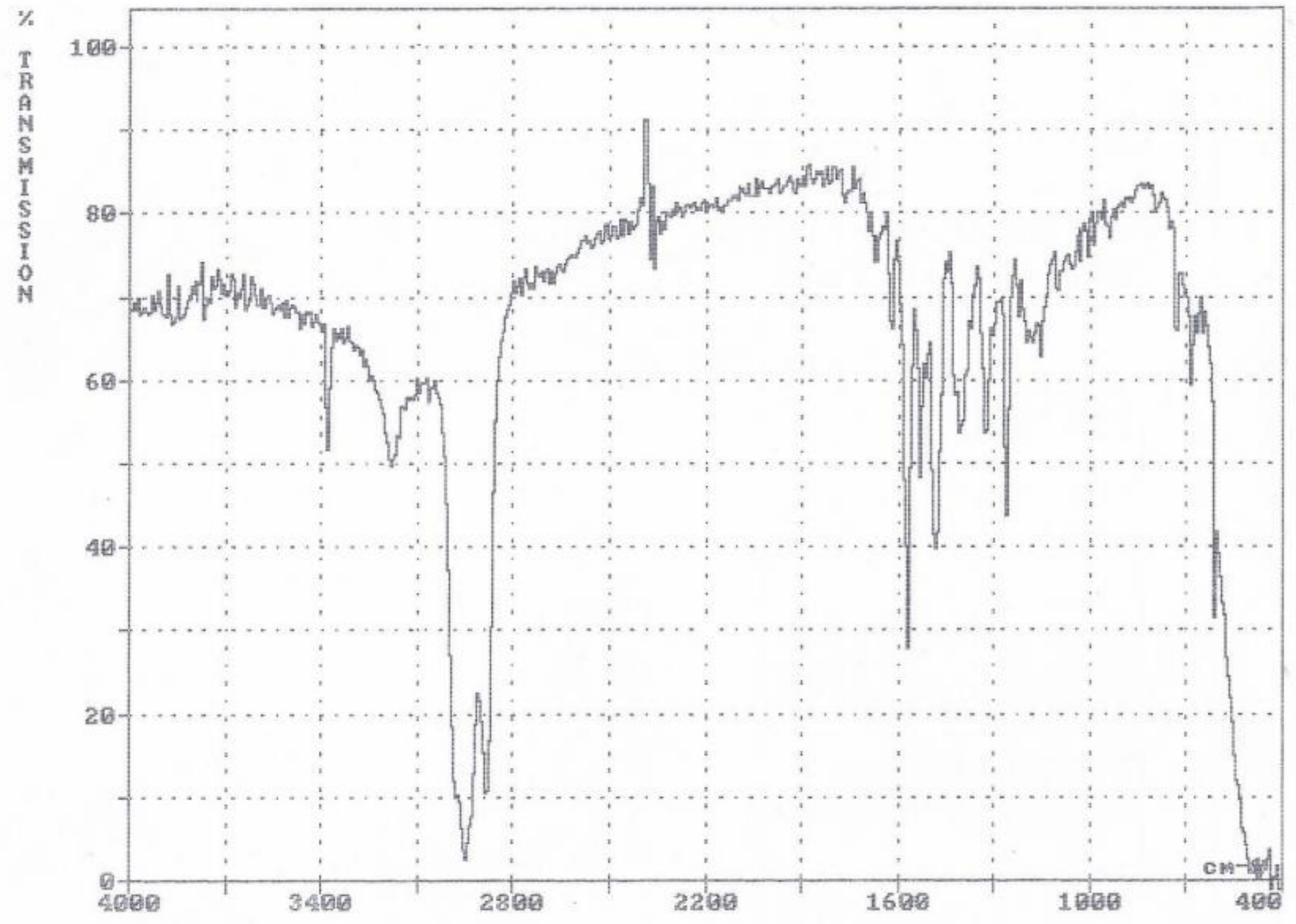

NMR ^{1}H of compound 4b

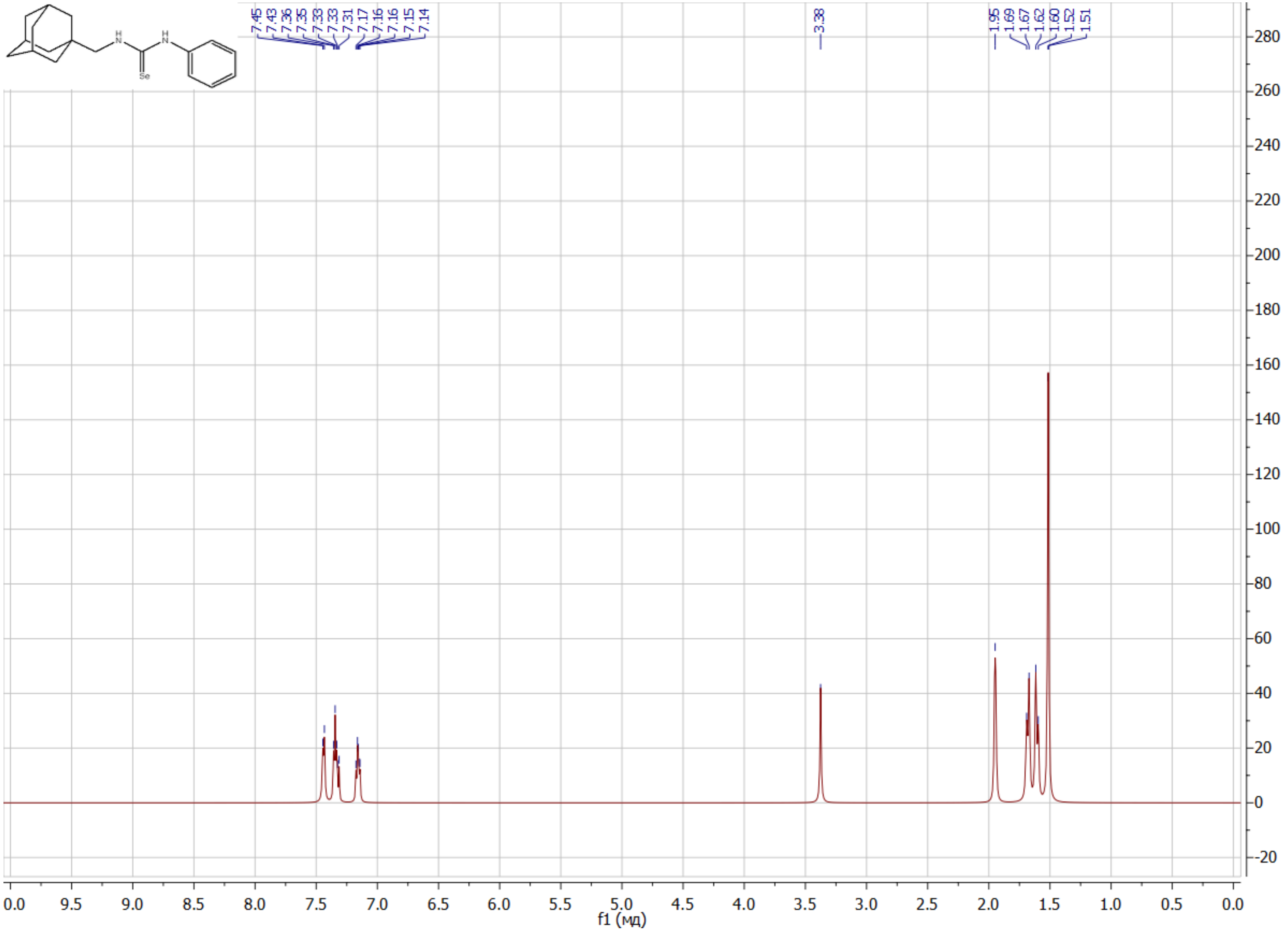

IR of compound 4b

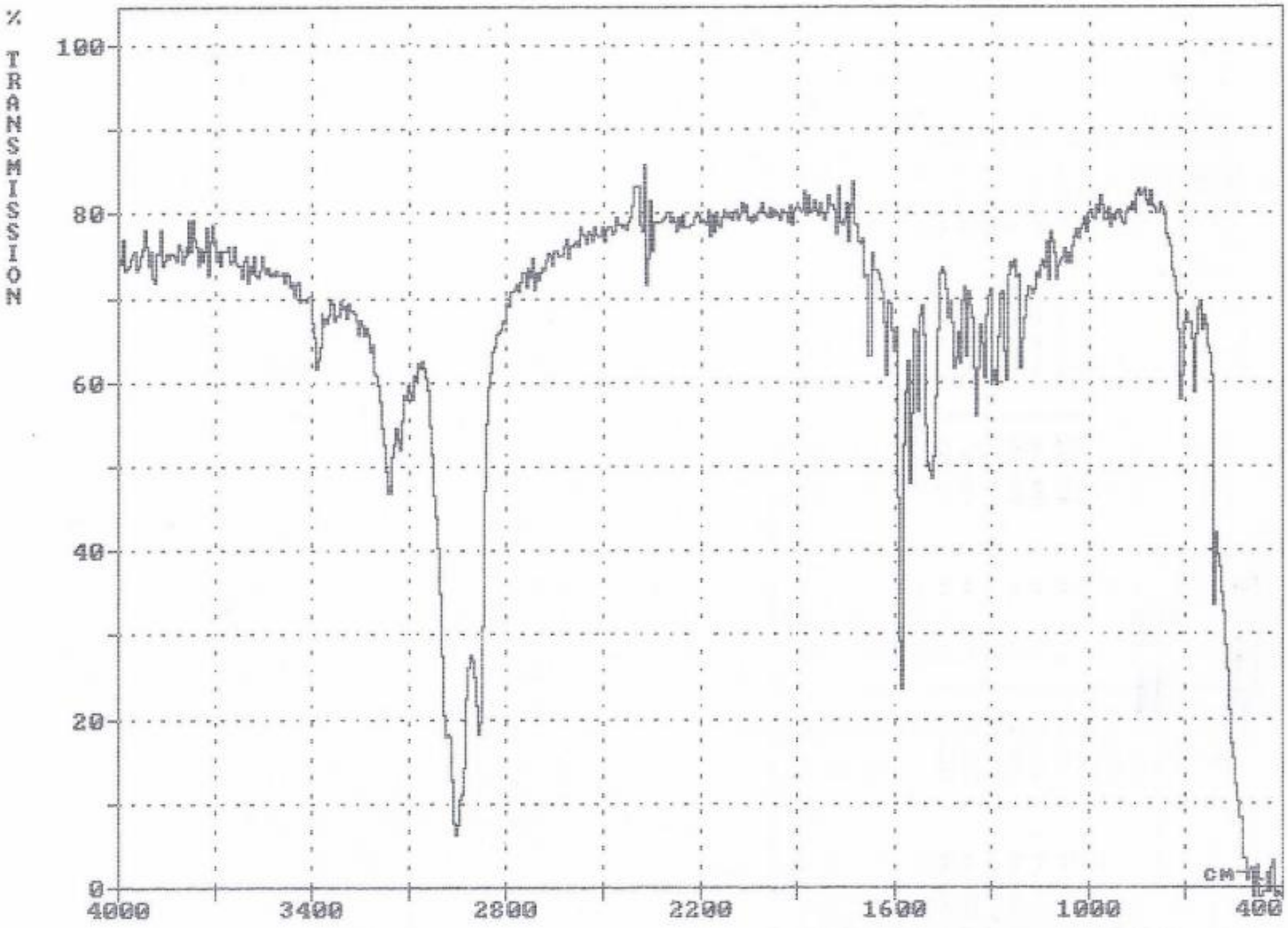

NMR ^{1}H of compound 4e

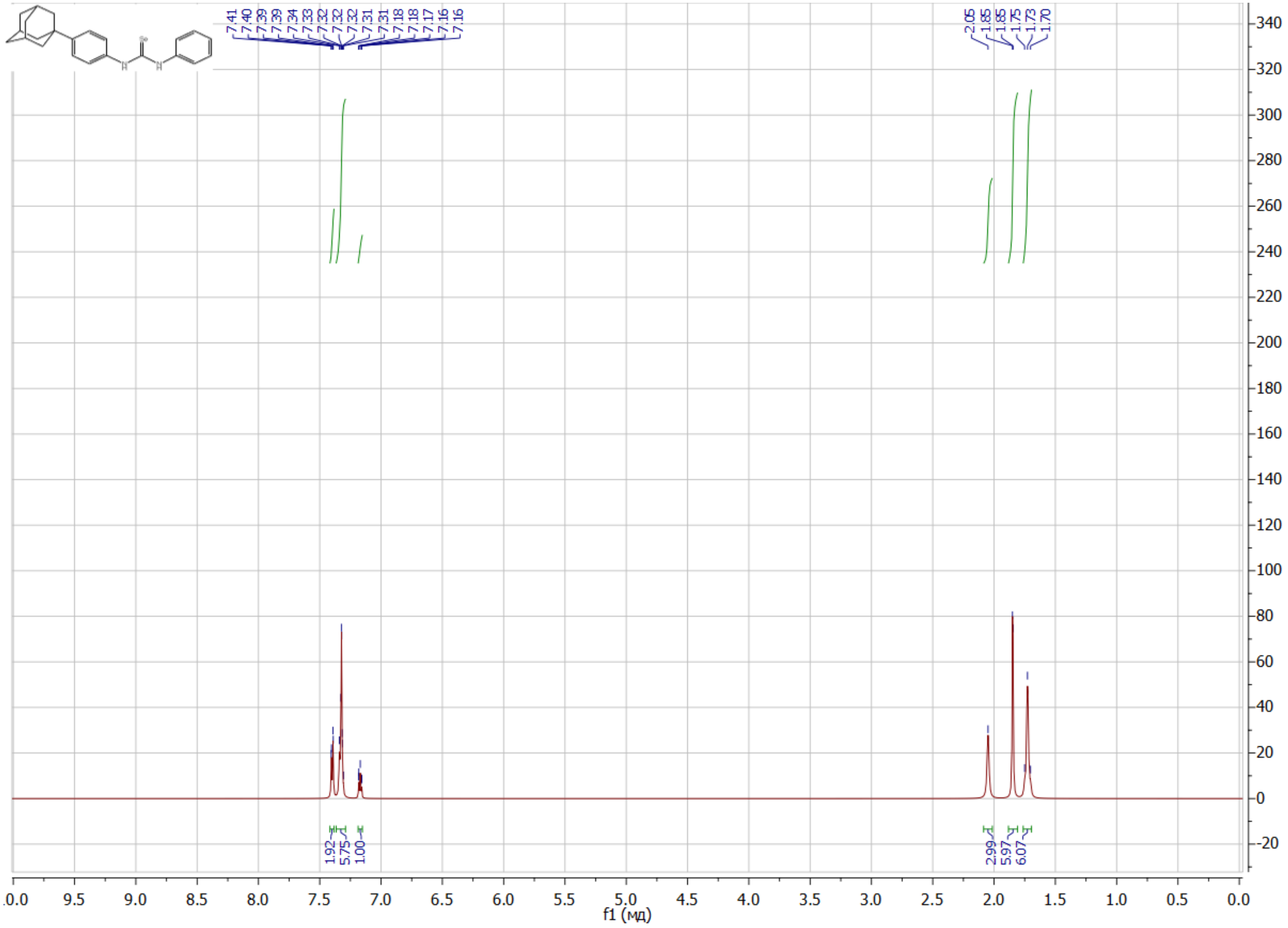

NMR ^{1}H of compound 4f

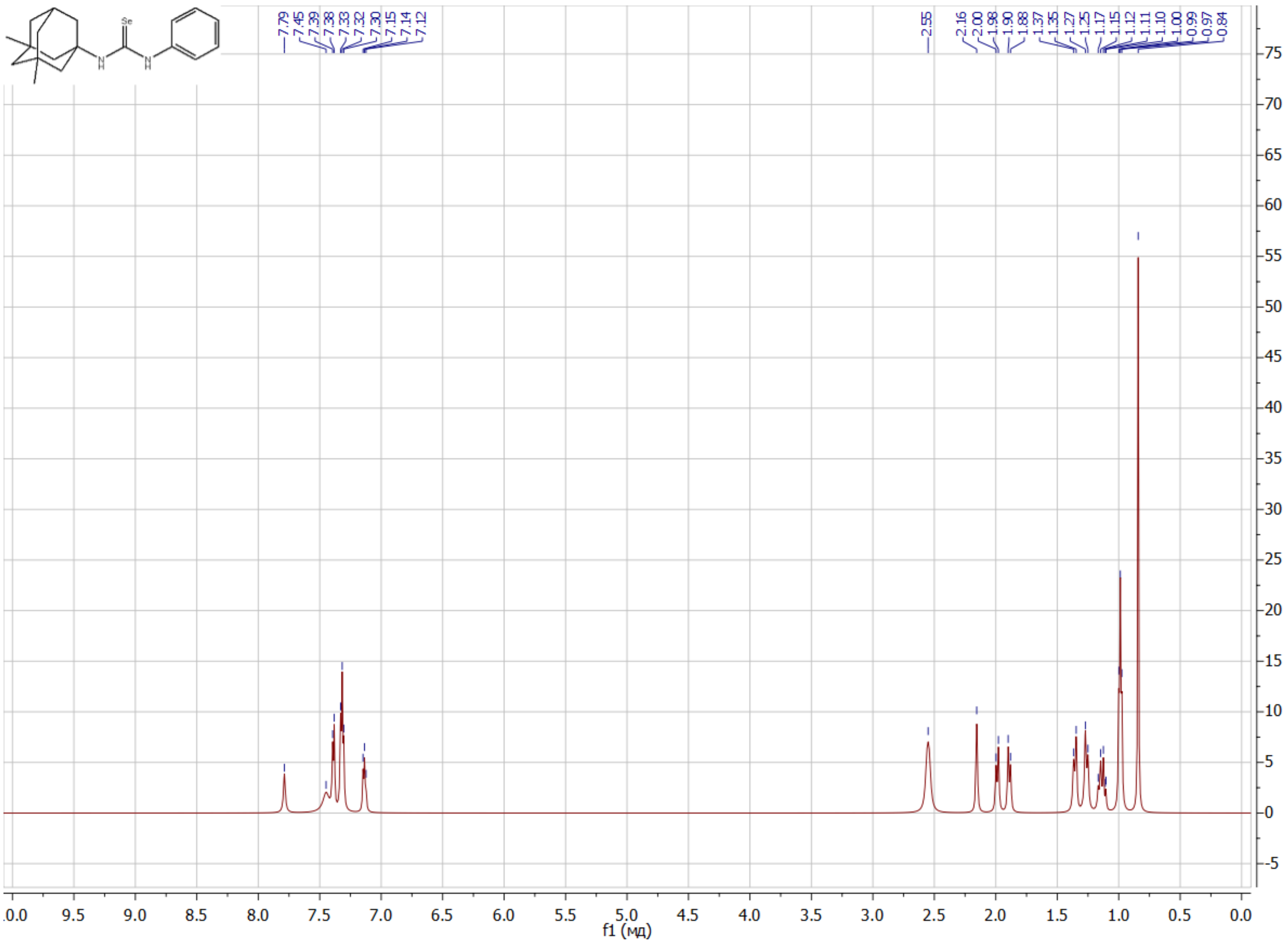

NMR ^{1}H of compound 4g

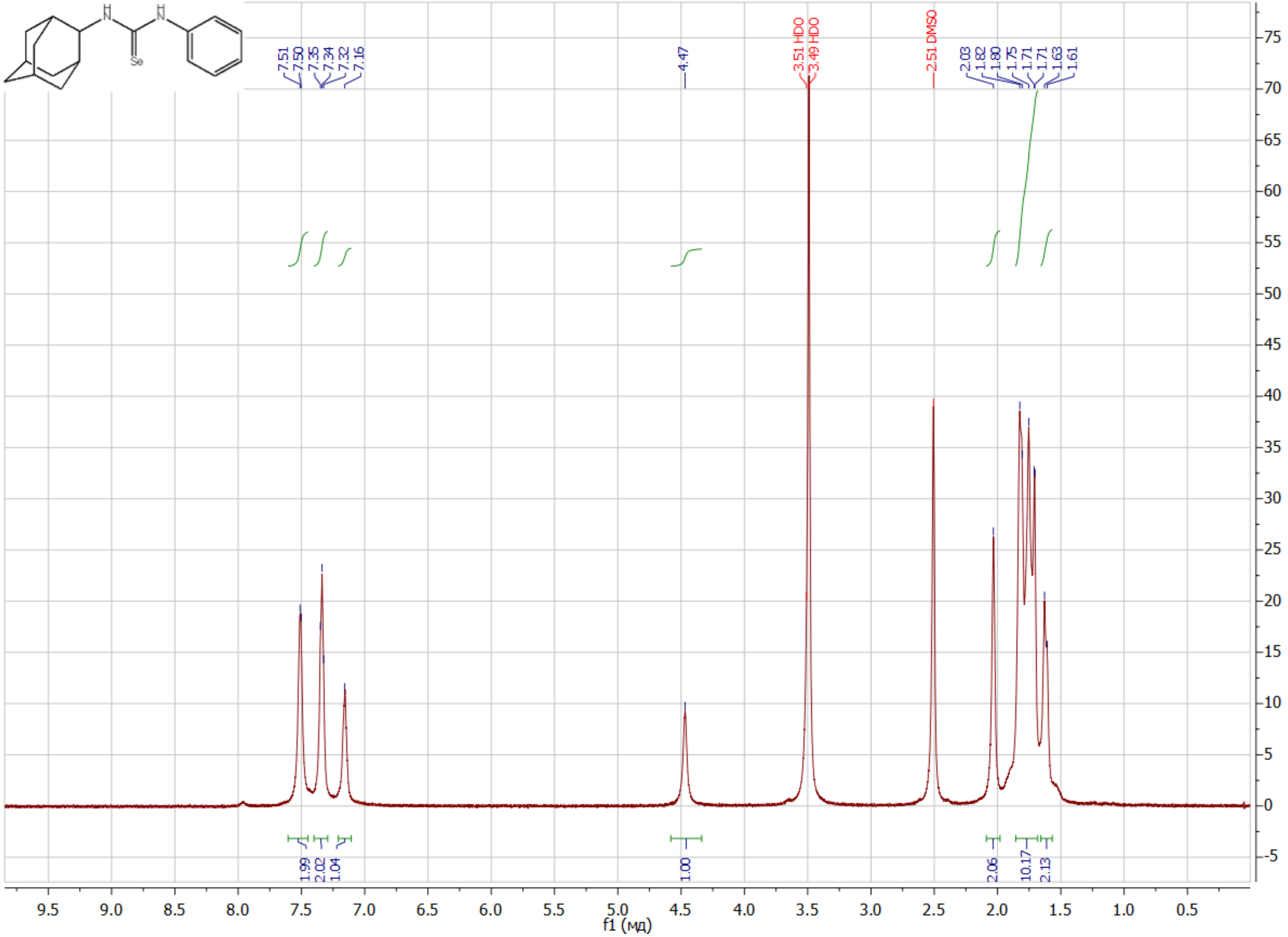

IR of compound 4g

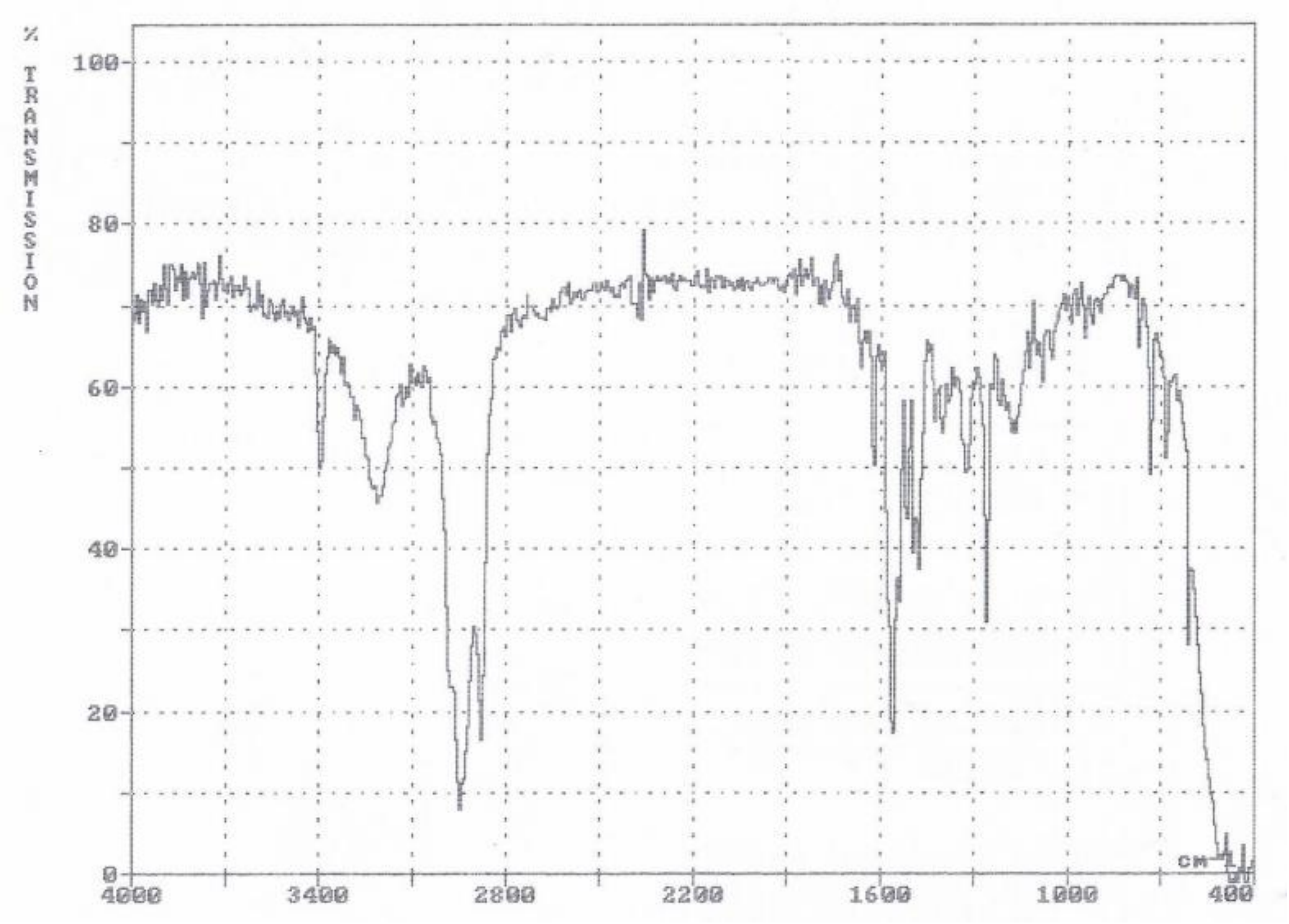

NMR ^{1}H of compound 5a

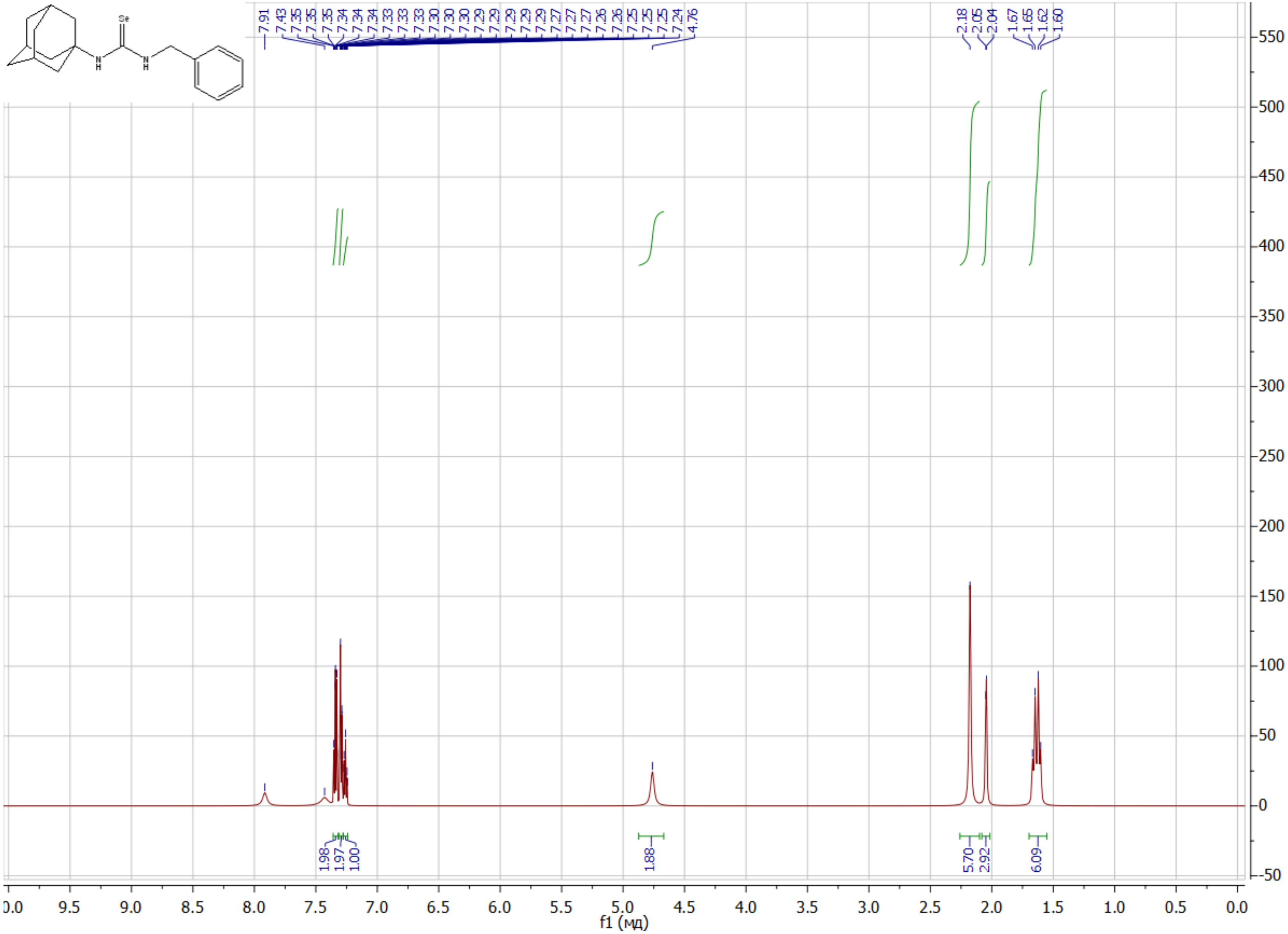

IR of compound 5a

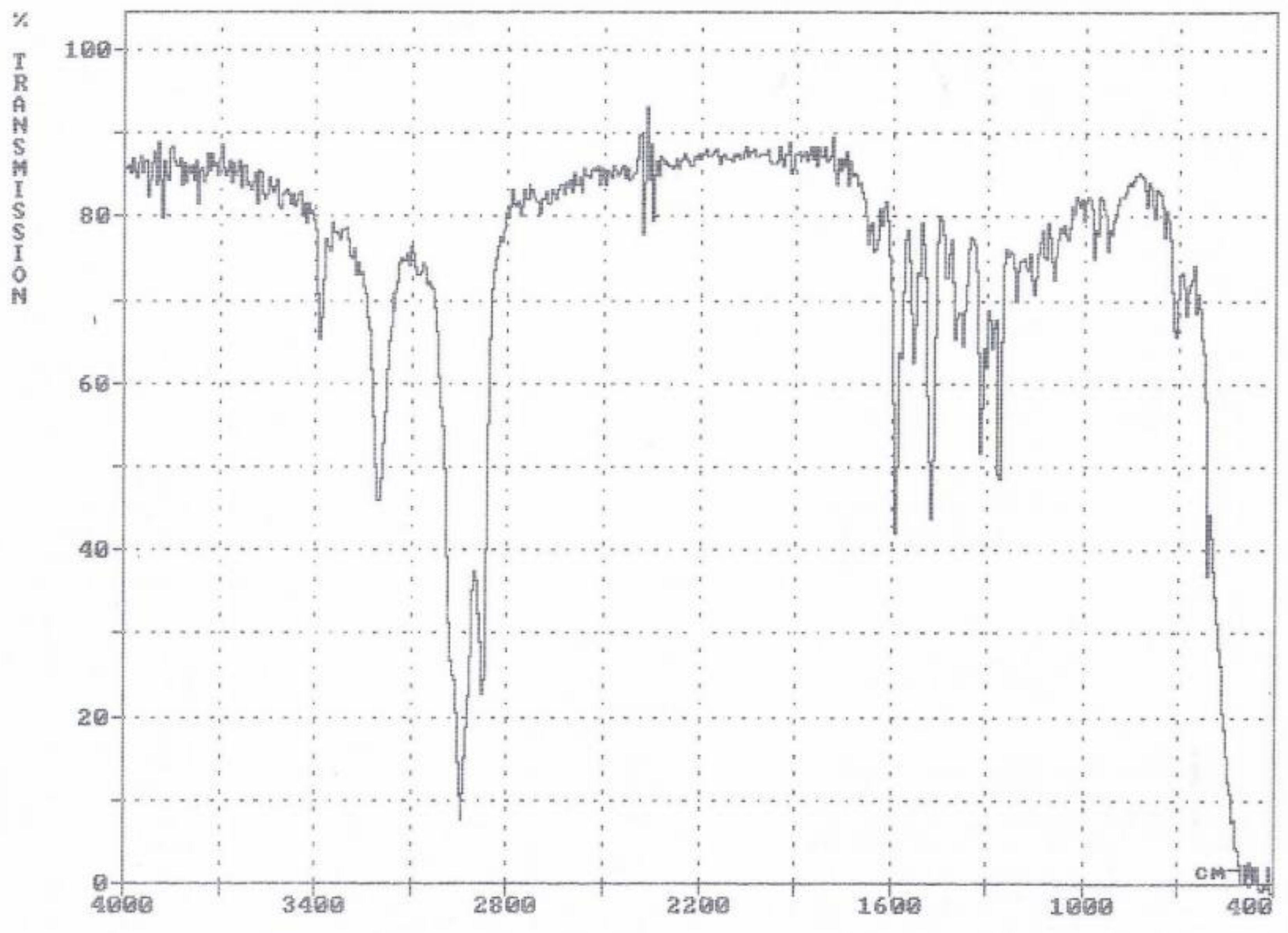

NMR ¹H of compound 5b

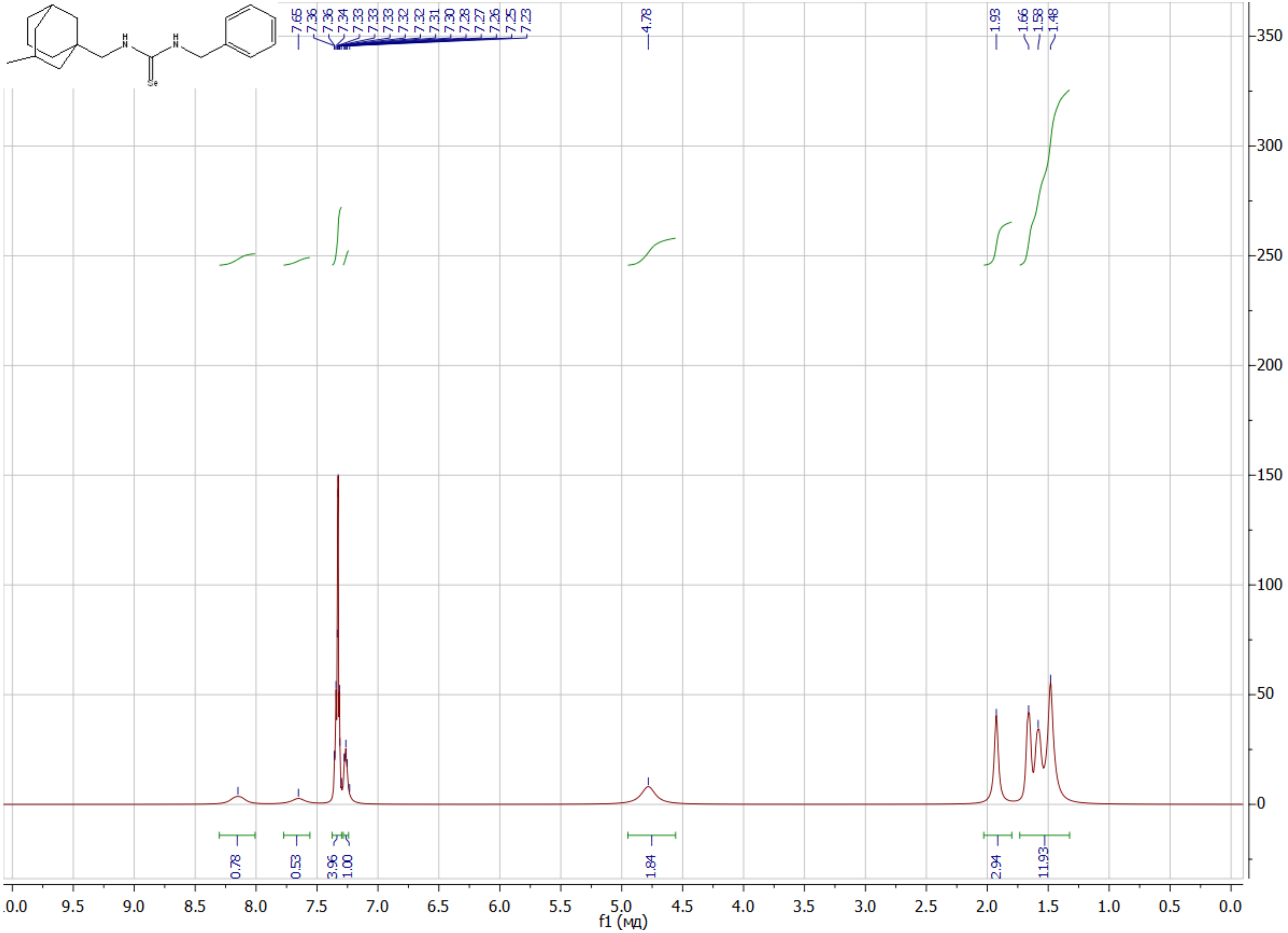

NMR ¹H of compound 5c

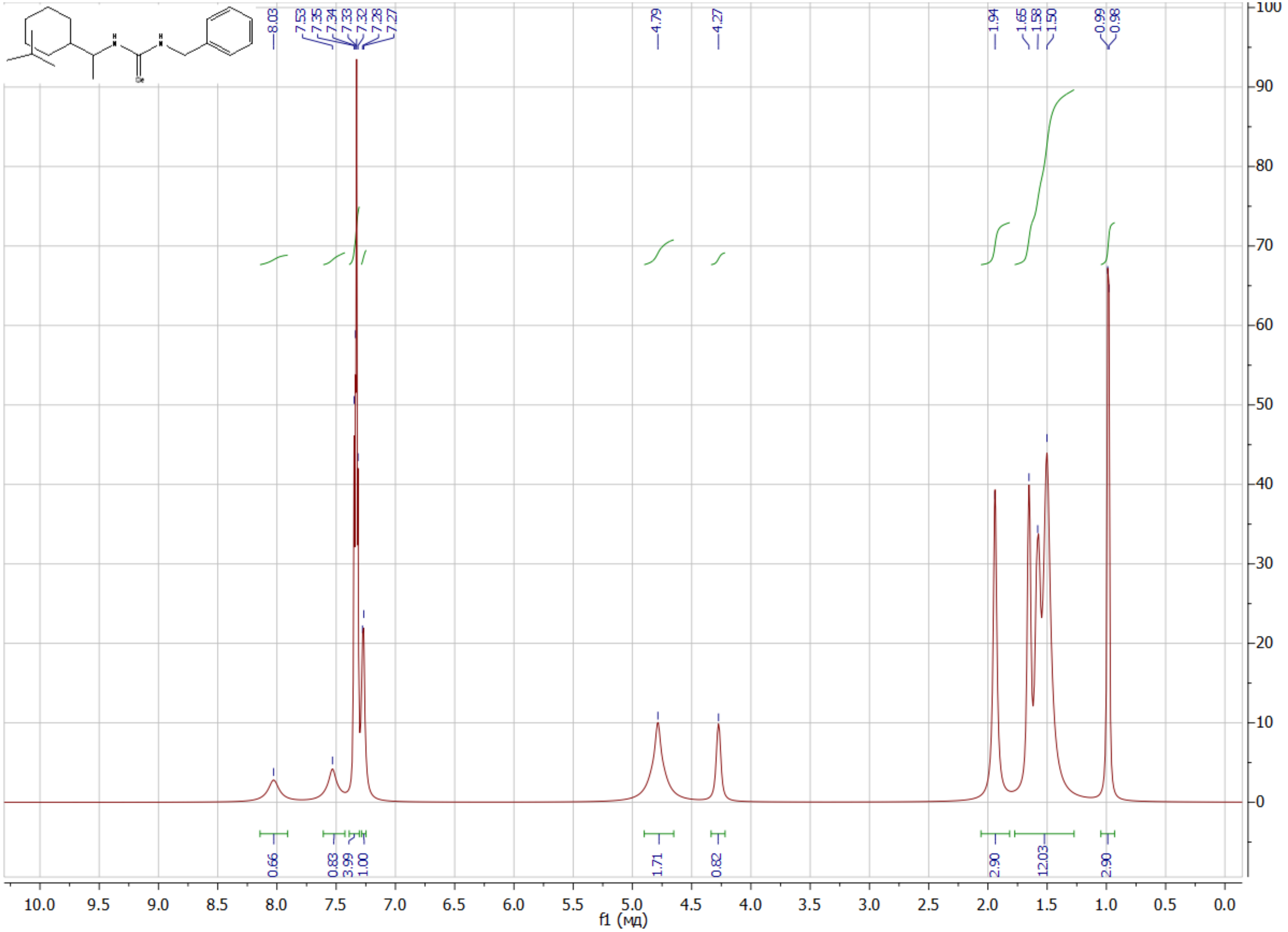

NMR ^{1}H of compound 5d

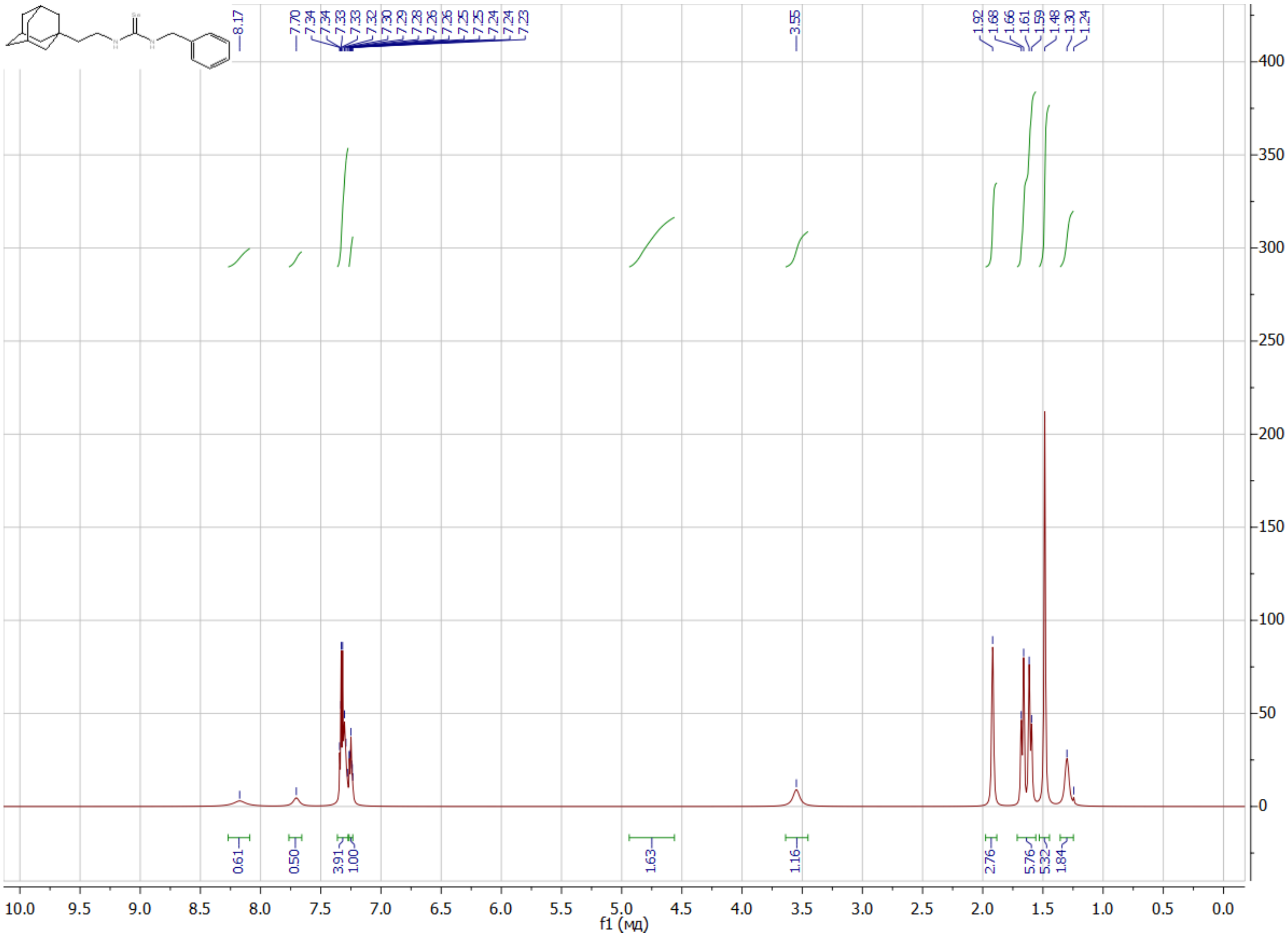

NMR ¹H of compound 5e

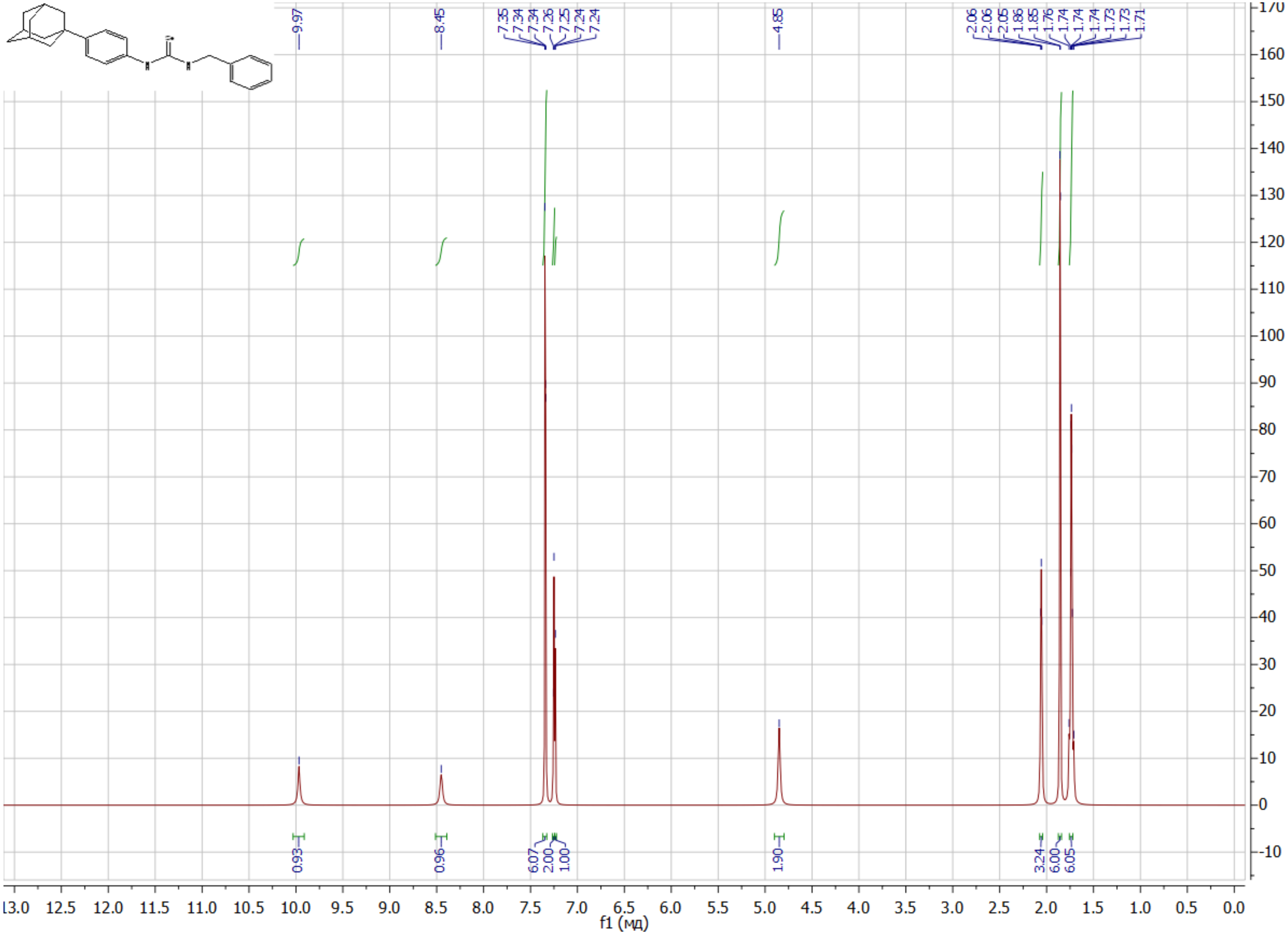

IR of compound 5e

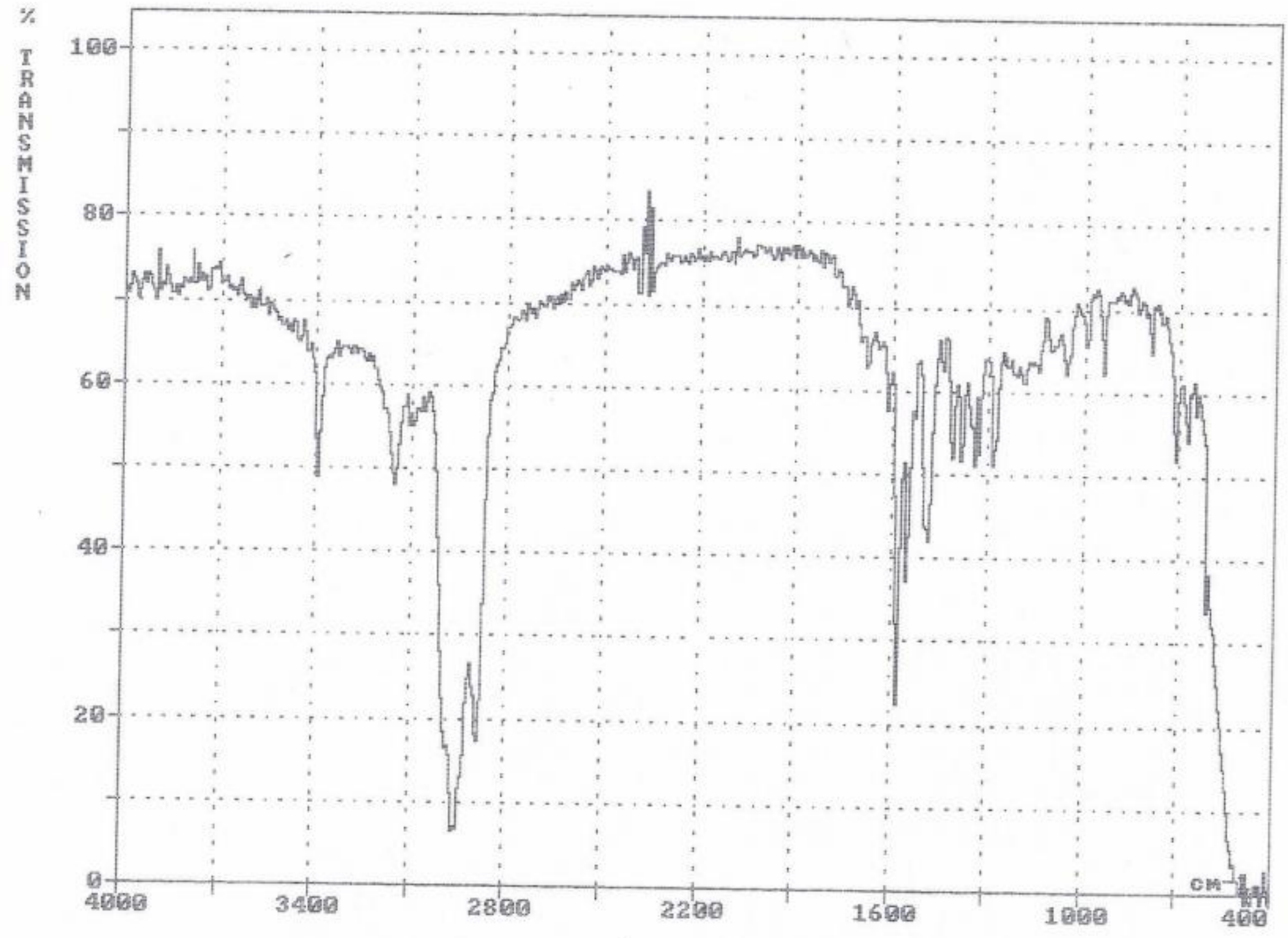

NMR ^{1}H of compound 5f

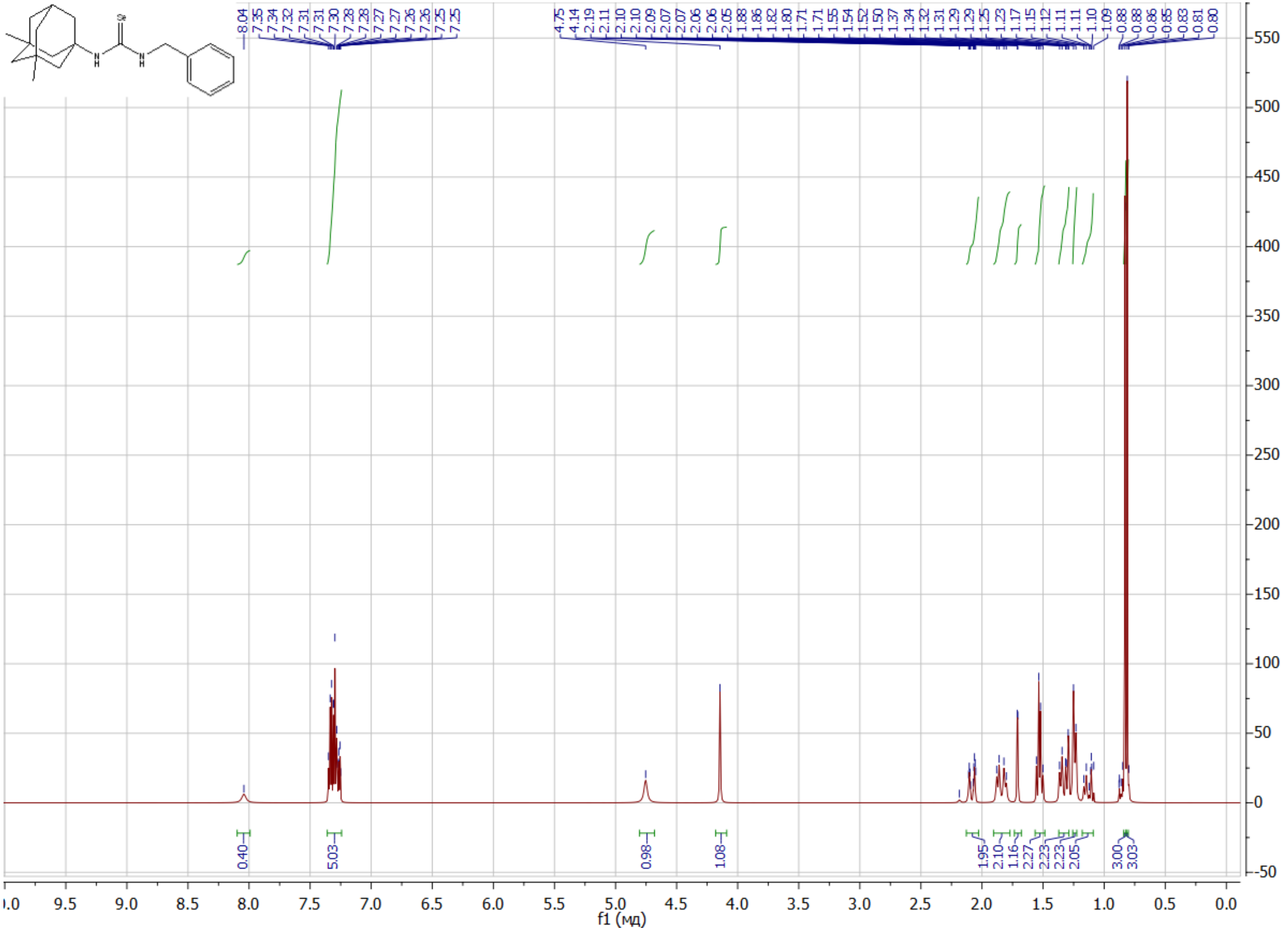

NMR ^{1}H of compound 5g

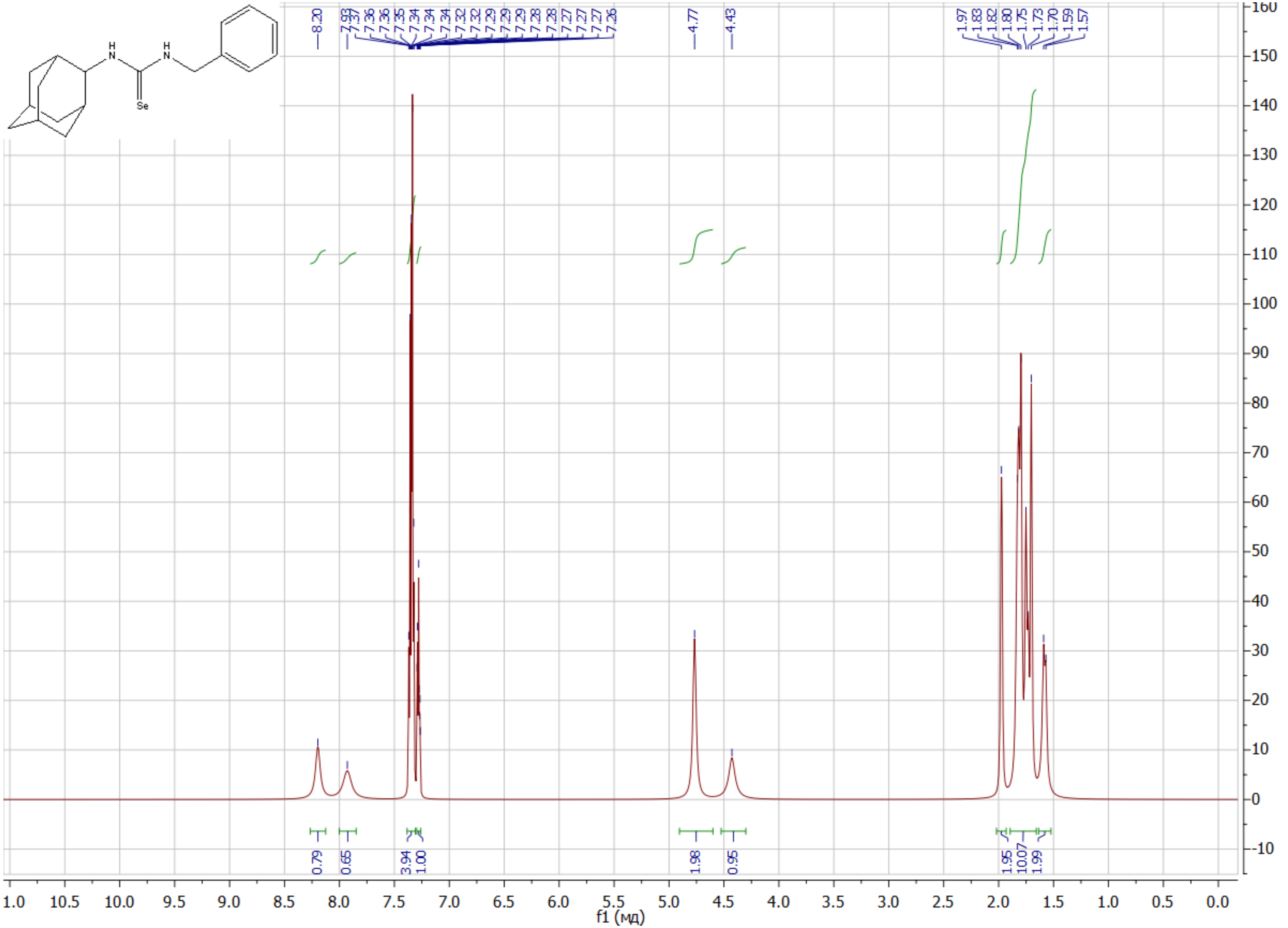

IR of compound 5g

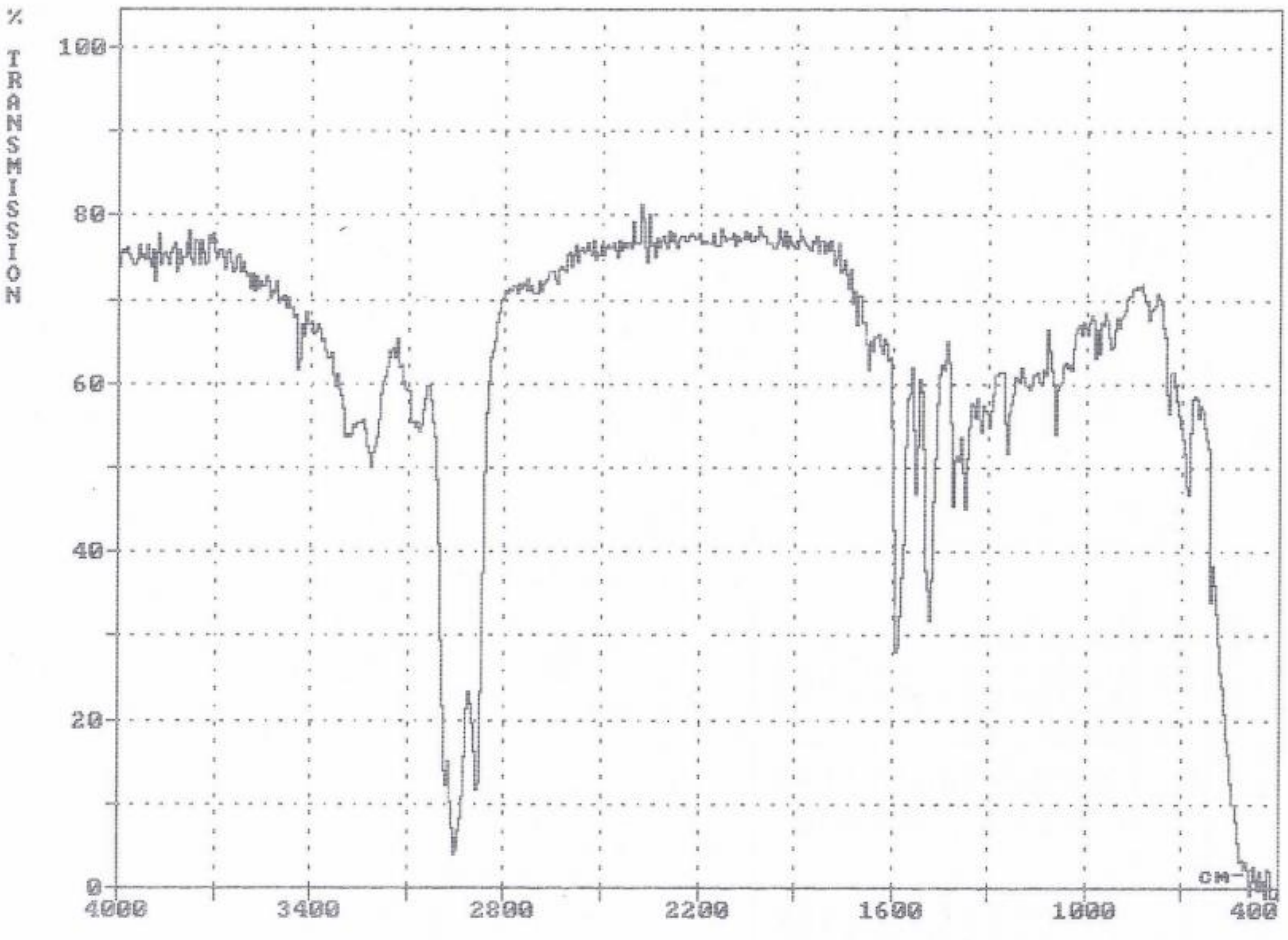

Supplement: Supplementary file 1 [file ijms-23-10710-s001.zip › ijms-1861255-supplementary.pdf]
